# CHILDHOOD MISFORTUNE PREDICTS LIFESPAN HEALTH INTO LATE ADULTHOOD

**DOI:** 10.1093/geroni/igz038.1273

**Published:** 2019-11-08

**Authors:** Nicholas A Turiano, Nicholas A Turiano, Kate A Leger, Patrick L Hill

**Affiliations:** 1 West virginia University, Morgantown, West Virginia, United States; 2 University of kentucky, Lexington, Kentucky, United States; 3 Washington University in St. Louis, St. Louis, Missouri, United States

## Abstract

Childhood misfortune encompasses a diverse set of negative early life experiences that have damaging effects on lifespan development. We extended this topic by examining how early life misfortunes predicted changes in measures of physical functioning (FUNC) and body mass index (BMI) in adulthood (ages 25-75). We used 3-wave data (N = 6,000) from the Midlife Development in the U.S. study across 20 years. Unconditional latent growth curve models (adjusting for age, sex, education) suggested significant (p < .05) mean-level change and variability in change for FUNC (Int = 1.47; Slope = 0.24) and BMI (Int = 26.71; Slope = 0.90). Higher levels of childhood misfortune (e.g., abuse, financial strain) significantly predicted worse FUNC (Int = 0.05; Slope = 0.02) and higher BMIs (Int = 0.24; Slope = 0.07) at baseline and steeper increases over time. Findings underscore the need to address adult health problems that emerge much earlier in life.

